# The duration of intervals on the oral cancer care pathway and implications for survival: a systematic review and meta-analysis

**DOI:** 10.3389/fpubh.2023.1183244

**Published:** 2023-08-08

**Authors:** Nicolás Francisco Fernández-Martínez, Dafina Petrova, Zuzana Špacírová, Rocío Barrios-Rodríguez, Mario Pérez-Sayáns, Luis Miguel Martín-delosReyes, Beatriz Pérez-Gómez, Miguel Rodríguez-Barranco, María José Sánchez

**Affiliations:** ^1^Instituto de Investigación Biosanitaria ibs.GRANADA, Granada, Spain; ^2^Escuela Andaluza de Salud Pública (EASP), Granada, Spain; ^3^CIBER of Epidemiology and Public Health (CIBERESP), Madrid, Spain; ^4^Department of Preventive Medicine and Public Health, University of Granada, Granada, Spain; ^5^Oral Medicine, Oral Surgery and Implantology Unit (MedOralRes), School of Medicine and Dentistry, University of Santiago de Compostela, Santiago de Compostela, Spain; ^6^ORALRES Group, Health Research Institute of Santiago de Compostela (IDIS), Santiago de Compostela, Spain; ^7^Department of Epidemiology for Chronic Diseases, National Center of Epidemiology, Instituto de Salud Carlos III, Madrid, Spain

**Keywords:** oral cancer, intervals, early diagnosis, TNM staging, survival

## Abstract

**Introduction:**

Previous studies measuring intervals on the oral cancer care pathway have been heterogenous, showing mixed results with regard to patient outcomes. The aims of this research were (1) to calculate pooled meta-analytic estimates for the duration of the patient, diagnostic and treatment intervals in oral cancer, considering the income level of the country, and (2) to review the evidence on the relationship of these three intervals with tumor stage at diagnosis and survival.

**Materials and methods:**

We conducted a systematic review with meta-analysis following PRISMA 2020 guidelines (pre-registered protocol CRD42020200752). Following the Aarhus statement, studies were eligible if they reported data on the length of the patient (first symptom to first presentation to a healthcare professional), diagnostic (first presentation to diagnosis), or treatment (diagnosis to start of treatment) intervals in adult patients diagnosed with primary oral cancer. The risk of bias was assessed with the Aarhus checklist.

**Results:**

Twenty-eight studies reporting on 30,845 patients met the inclusion criteria. The pooled median duration of the patient interval was 47 days (95% CI = 31–73), *k* = 18, of the diagnosis interval 35 days (95% CI = 21–38), *k* = 11, and of the treatment interval 30 days (95% CI = 23–53), *k* = 19. In lower-income countries, the patient and treatment intervals were significantly longer, and longer patient intervals were related to later stage at diagnosis. In studies with a lower risk of bias from high-income countries, longer treatment intervals were associated with lower survival rates.

**Conclusion:**

Interval duration on the oral cancer care pathway is influenced by the socio-economic context and may have implications for patient outcomes.

## 1. Introduction

Oral cancer is the most incident type of head and neck cancer. In 2020, lip and oral cavity cancer together accounted for an estimated 377,713 new cases worldwide (40.5% of all new cases of head and neck cancer) (1). Its global annual age-standardized rate (ASR-W) is 4.1 cases per 100,000 population, showing broad variation across regions. Melanesia and South-Central Asia present the highest ASR, while the lowest is found in Western Africa (1). Although therapeutic advances have improved 5-year overall survival in the last decades up to about 40–60% (2, 3), oncologic outcomes remain poor, especially in patients with advanced disease (4). Therefore, there is a need to develop strategies aimed at improving survival for patients with oral cancer.

The timeliness of oral cancer diagnosis and treatment is thought to play a crucial role in patients’ outcomes. This is usually assessed by estimating the time elapsed between key events in the cancer care pathway described in the Model of Pathways to Treatment proposed by Andersen et al. (5, 6). In particular, three intervals describe the time elapsed between the start of symptoms and treatment initiation. The patient interval comprises the time from symptom onset to first presentation to a healthcare professional; the diagnostic interval, from first presentation to diagnosis; and the treatment interval, from diagnosis to start of treatment.

Delays in oral cancer diagnosis are common because of patient and healthcare system factors. Patient factors include health-related behaviors and lack of awareness (7), while inadequate healthcare access constitutes the main healthcare system factor (8). In addition, comorbidity and the primary treatment modality (e.g., surgery or radiotherapy) can both influence the time elapsed between diagnosis and treatment (9).

Available evidence suggests that delays in oral cancer diagnosis and treatment are associated with worse patient outcomes. In particular, longer time intervals to diagnosis have been associated with advanced tumor stage at diagnosis in some studies (10). In turn, tumor stage is strongly associated with survival (11) and the degree of spread to regional lymph nodes (pN) is one of the main predictors of survival in cancer of the oral cavity (12). Furthermore, longer treatment intervals have been associated with lower survival (13). A U-shaped association indicating higher mortality rates in patients with the shortest and longest times to treatment has been described (14). However, previous research on time intervals in oral cancer is characterized by heterogeneity in study designs, particularly concerning the definition of intervals, which has limited our understanding of their impact (15).

Beyond clinical aspects, cancer intervals are also related to social factors. A recent meta-analysis estimated that patient intervals in head and neck cancers are twice as long in low- and middle-income countries (LMICs) compared to high-income countries (16). To further complicate matters, the incidence of oral cancer in countries with low or medium human development index (HDI) is twice as high as that in their high or very high HDI counterparts (17), suggesting that socioeconomic factors partially explain geographical disparities (18). Additionally, socioeconomic deprivation has also been identified as a determinant of survival in oral cancer (19).

Previous systematic reviews have documented a large variation in the duration of different intervals on the cancer care pathway and their relationship with factors such as tumor stage, symptoms, and survival (15, 20–22). However, socioeconomic factors have seldom been considered and no previous review has offered a meta-analysis of the three main intervals comprising the cancer care pathway. Thus, the first objective of this review was to calculate pooled meta-analytic estimates of the duration of the patient, diagnostic, and treatment intervals in oral cancer, considering the income level of the country. The second objective was to systematically compile and synthesize the evidence on the relationship of these three intervals with tumor stage at diagnosis and survival.

## 2. Materials and methods

The PRISMA 2020 statement for conducting and reporting systematic reviews was followed (23). This study is based on a larger review of diverse cancer sites, including oral cancer (pre-registered protocol CRD42020200752) (16).

### 2.1. Eligibility criteria

For the larger review, studies reporting data on the length of the patient, diagnostic, or treatment intervals for any cancer site in adult patients (defined as mean sample age ≥ 30 years) presenting with symptomatic confirmed primary cancers were considered. The following were excluded: (a) articles not reporting the results of original studies, (b) qualitative studies not reporting interval duration in a way possible for analysis, (c) studies reporting on children, adolescents, or young adults, (d) studies reporting on patients with cancer diagnosed accidentally or through screening, (e) studies reporting on patients with relapsed cancer, (f) studies in languages not understood by the research team (i.e., languages other than English, Spanish, French, Portuguese, German, Dutch, Bulgarian, and Slovak), and (g) studies not reporting the sample size. If studies reported interval duration for periods after the start of the COVID-19 pandemic and authors discussed its potential effects on the intervals reported, only intervals prior to the pandemic were considered. The larger review included multiple cancer sites (e.g., breast cancer), which extend beyond the scope of this work; therefore, in the current review, only articles reporting data on patients with oral cancer, defined as lip and oral cavity cancer (ICD-10 codes C00-C06), were further selected.

According to the Aarhus Statement on early cancer diagnosis (6), the definitions of the intervals were the following: the time elapsed between the date of the first symptom and the date of the first presentation to a healthcare professional (patient interval), the time elapsed between the date of first presentation to a healthcare professional and the date of diagnosis (diagnostic interval), and the time elapsed between the date of diagnosis and the date of the start of treatment (treatment interval). Studies had to report, at a minimum, the median or mean duration of the interval in days, weeks, or months, and the number of patients, in order to be included.

### 2.2. Data sources and search strategy

A broad search strategy in MEDLINE (using the Ovid platform), Embase, and Web of Science (WOS)-Core Collection was designed and conducted by a librarian (the search strategy can be found in Supplementary Data S1). Moreover, to identify gray literature, Google Scholar, EThOS, OpenGrey, and ProQuest Dissertations & Theses were searched. Last, backward citation searching (e.g., references from articles deemed as relevant were also reviewed) was conducted to identify additional studies. The search strategy included articles published from January 2009 to May 2022. The date of start was chosen to match the date of publication of the Olesen Model (24) and the Model of Pathways to Treatment (25), two seminal articles on the intervals throughout the cancer care continuum. No restrictions were imposed by language or country.

### 2.3. Article selection

The Covidence software^1^ was used to manage the systematic review. Blind screening of 26% of the abstracts retrieved was independently performed by two reviewers. Agreement was satisfactory against the pre-established criterion of >90%, with agreement rates ranging from 87 to 100%; disagreements were discussed among the reviewers. Afterward, abstract screening was continued individually.

Full text screening was performed by two reviewers blinded to each other’s decisions. Disagreements were documented and resolved by discussion or by a third reviewer. Reasons for exclusion were further documented.

### 2.4. Data extraction

For each study, the following were recorded: year of publication, period of data collection, country, total number of patients with oral cancer, study design, study setting, data sources (i.e., questionnaires/interviews, medical records and/or specialized large databases), inclusion and exclusion criteria, type of interval (s) studied, availability of data on tumor stage, and availability of data on survival.

For each interval, statistical data recorded if available (in days) included: median, interquartile range, minimum, maximum, mean, standard deviation (SD), sample size (N)–considering only patients with data on the duration of any of the intervals of interest–, country, year of start and end of data collection, mode of diagnosis confirmation, and modality of the first treatment if specified (concerning the treatment interval).

According to the 8th edition of the Union for International Cancer Control (UICC) TNM classification, tumor stage ranges from disease localized to the organ of origin (I and II) to locally extensive spread to regional lymph nodes (III) and distant metastasis (IV) (26). For the tumor stage, the proportion of patients with each UICC/AJCC stage (I, II, III, IV) and its association with the length of the intervals of interest were recorded.

For survival, the type (i.e., overall, disease-specific, recurrence-free, and/or relative survival) and measure of the outcome (i.e., the survival rate at a given number of years and/or median survival), along with its association with the length of the intervals of interest were recorded.

### 2.5. Risk of bias

The risk of bias concerning the intervals on the cancer treatment pathway in each study was evaluated using a short form of the “Aarhus statement checklist” (6) developed for studies quantifying such intervals. The checklist contains questions regarding interval definitions, measurement, healthcare context, use of theoretical frameworks, discussion of validity, and biases, among others (Supplementary Data S2 contains the Aarhus checklist). It was completed independently by two reviewers and disagreements were resolved by a third reviewer. Studies with scores below 25% were considered as high risk, studies with scores of 75% and above as low risk, and the remainder as intermediate risk [the Aarhus statement does not define risk thresholds, so a previously used categorization was followed (16)].

### 2.6. Synthesis of results

To estimate the pooled duration of the patient, diagnostic, and treatment intervals, a meta-analysis was conducted using the “metamedian” package (27) in R software version 4.1.1 (28), based on the “median of medians” method suitable for heterogeneous data. Study-specific estimates were combined in a pooled median and 95% confidence intervals (CI) were calculated.

This process was done for all studies together and stratified by income level of the country according to the gross national income (GNI). Countries were classified as “low-income” with GNI of less than $1,046, “lower-middle income” with GNI between $1,046 and $4,095, “upper-middle income” between $4,096 and $12,695, and “high income” with GNI of more than $12,695 per capita, following the thresholds agreed on 2021 for fiscal year 2022 (29).

If the median is not reported, the mean can be used instead in the “metamedian” package (27). In our case, means were imputed as medians in 6, 0, and 37% of studies analyzing the patient, diagnostic, and treatment interval, respectively. Yet, using means may introduce bias when the mean is not a sufficiently good approximation of the median. For this reason, as sensitivity analysis, we estimated the pooled median duration of intervals after excluding studies that only reported means. We also estimated the pooled median duration after excluding studies with a high risk of bias.

To summarize results on the relationship between the intervals of interest and tumor stage and survival, we performed a qualitative synthesis of results. Considerable heterogeneity among studies was observed regarding how tumor stage and survival were reported, which prevented us from conducting a quantitative synthesis.

## 3. Results

### 3.1. Description of studies

**Figure 1:**
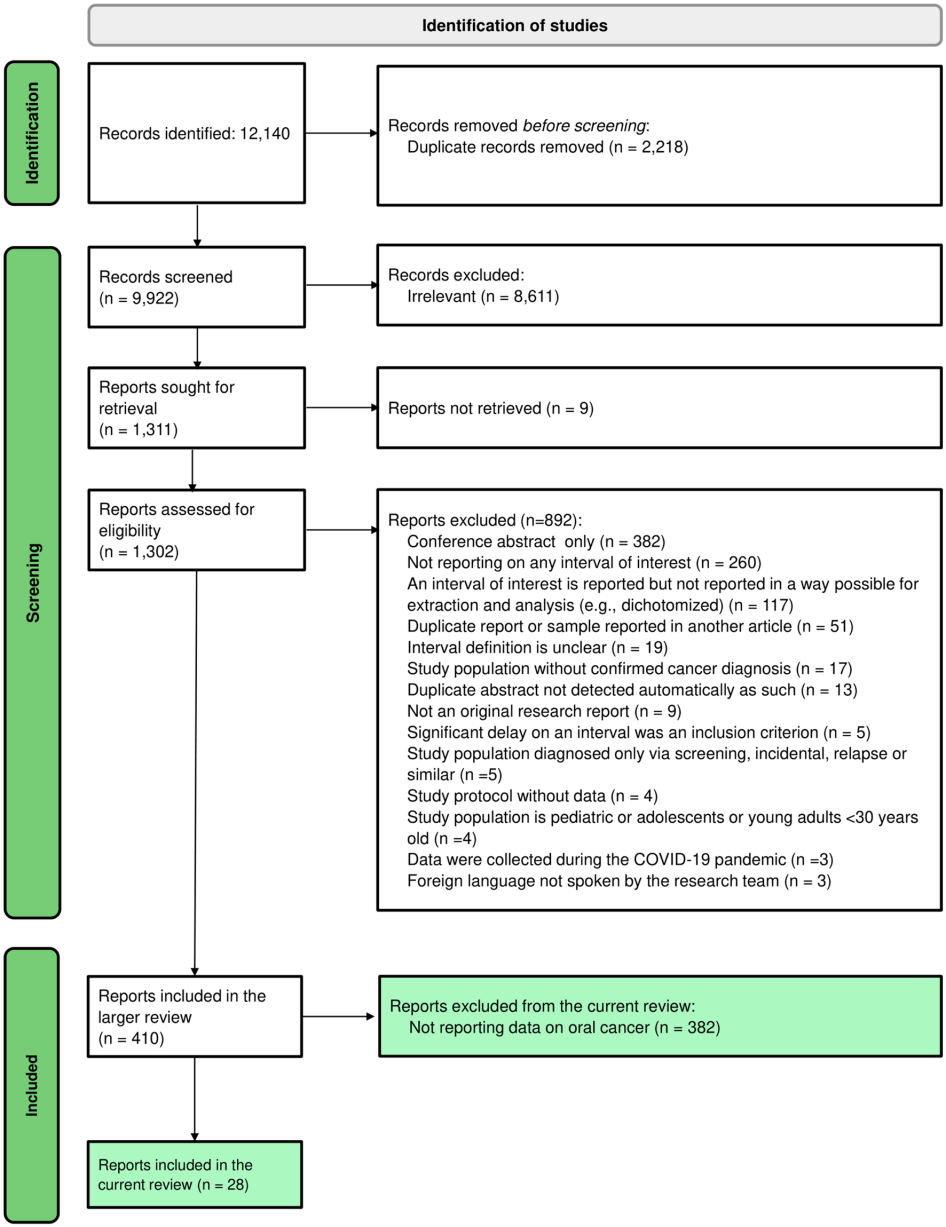
PRISMA flow diagram of the study selection process.

The initial search retrieved a total of 9,922 records after the removal of duplicates, of which 410 articles met the eligibility criteria and were included in the larger review. Among them, 28 studies reported at least one of the intervals of interest in patients with oral cancer and were thus included in the current review (30–57). Figure 1 shows the flow diagram of the article selection process.

Table 1 shows the most relevant information about each article (further information is presented in Supplementary Data S3, available on the Open Science Framework). The studies included were conducted in 16 different countries, Australia and India being the most frequent with three articles each; the majority of studies reported data from high-income countries (*n* = 17). Most studies were retrospective (*n* = 13) or cross-sectional (*n* = 13).

**Table 1:** Detailed summary of studies included in the systematic review.

References	Date of publication/Period of data collection	Country	N	Design	Inclusion and exclusion criteria	Quality (risk of bias)	Interval estimates	Stage at diagnosis	Survival
Gao and Bin (30)	2009/2005–2006	China	102	Cross-sectional	Inclusion: patients ≥18 years with oral squamous cell carcinoma (ICD codes not specified) Exclusion: patients who could not provide a reliable history	38% (Medium)	PI: median = 49 d DI: median = 49 d	Not studied	Not studied
Sargeran et al. (31)	2009/2004–2006	Iran	100	Cross-sectional	Inclusion: patients diagnosed with primary oral squamous cell carcinoma (ICD-10 codes C01-C06) who were referred to one of three university hospitals in Teheran Exclusion: patients with a previous history of other cancers and patients who failed to sign the informed consent for participation	64% (Medium)	PI: median = 60 d DI: median = 30 d	I-II 39% III-IV 61% Longer patient intervals were associated with more advanced stages (for stages III-IV: OR = 5.6[95%CI = 1.8–17.3] when PI was categorized into ≤60 and > 60 days) Longer diagnostic intervals were associated with more advanced stages (for stages III-IV: OR = 3.8[95%CI = 1.2–9.4] when DI was categorized into ≤30 and > 30 days)	Not studied
Teppo and Alho (32)	2009/1986–1996	Finland	62	Retrospective cohort	Inclusion: all patients with histologically verified squamous cell carcinoma of the larynx, pharynx, and anterior mobile tongue (ICD-9 and ICD-10 codes 161, C32, 146–148, C09-C11, C13, 141, C02) diagnosed and treated at Oulu University Hospital Exclusion: none	67% (Medium)	PI: median = 42 d DI: median = 21 d	I 13% II 35% III 40% IV 11% No significant association with the patient or diagnostic interval	Not studied
Kolude et al. (33)	2013/1991–2010	Nigeria	169	Cross-sectional	Inclusion: persons with oral and maxillofacial cancers (including carcinomas, sarcomas, and lymphomas; ICD codes not specified) retrieved from the archival records Exclusion: none	14% (High)	TI: median = 103 d in women, median = 55 d in men	I-II 16% III-IV 84% Association with the treatment interval not studied	Not studied
Esmaelbeigi et al. (34)	2014/2009–2010	Iran	206*	Cross-sectional	Inclusion: all patients with incident cancer of the tonsils, tongue, mouth, and oropharynx (the most common histology (92%) was squamous cell carcinoma; ICD-10 codes C01-C06, C09-C10) who were admitted to the Cancer Institute of Iran for surgical treatment Exclusion: cases of recurrent cancer	17% (High)	PI: median = 45 d	Only available for the total sample (including oropharyngeal cancer): I-II 29% III-IV 71% Association with the patient interval not studied	Not studied
Zhang et al. (35)	2015/1998–2010	Canada	554	Cross-sectional	Inclusion: patients >18 years residing in Alberta with biopsy-proven oral cavity squamous cell carcinoma (ICD codes not specified) who were treated in Alberta with curative intent Exclusion: patients with previous head and neck cancer, patients refusing the prescribed treatment, and patients with incomplete data	38% (Medium)	PI: mean = 49 d in urban areas, mean = 53 d in rural areas, mean = 53 d in intermediate areas	I 37% II 23% III 5% IV 34% Association with the treatment interval not studied	5-year overall survival = 65% 5-year disease-specific survival = 67% Association with the treatment interval not studied
Baishya et al. (36)	2015/2014	India	141	Cross-sectional	Inclusion: patients diagnosed with head and neck cancer (including histological, radiological, or clinical confirmation; ICD-10 codes C00-C14, C32) attending a regional cancer center in North East India Exclusion: patients who were unable or unwilling to participate in the study	25% (Medium)	PI: median = 90 d in tongue cancer, median = 60 d in mouth cancer, median = 90 d in lip cancer	Not studied	Not studied
Chiou et al. (37)	2016/2007	Taiwan	2,703	Retrospective cohort	Inclusion: patients from Taiwan who were diagnosed with oral cancer (ICD-O codes C060, C069) Exclusion: patients with an initial treatment date more than 365 days after the date of microscopic examination and patients with incomplete or erroneous data	86% (Low)	TI: median = 18 d	I 21% II 20% III 17% IV 41% No significant association with the treatment interval	Median survival = 1,573 d Mean survival = 1,363 d Longer treatment intervals were associated with lower survival time (*p*-value of log-rank test = 0.037 when TI was categorized into <21 and > 21 days)
Kaing et al. (38)	2016/2008–2010	Australia	101	Retrospective cohort	Inclusion: all patients treated at the Royal Melbourne Hospital for a first-time histological diagnosis of oral squamous cell carcinoma (ICD codes not specified) Exclusion: none	14% (High)	TI: median = 30 d	I 36% II 24% III 13% IV 28% Association with the treatment interval not studied	2-year overall survival = 86% Association with the treatment interval not studied
Fujiwara et al. (39)	2017/1998–2011	United States	4,868	Retrospective cohort	Inclusion: patients diagnosed or first treated for primary oral cavity squamous cell carcinoma (ICD codes not specified) with recorded pathologic staging at an NCDB site, and treated by surgery, radiotherapy, or chemotherapy. Exclusion: patients with multiple cancers, patients not treated at the reporting facility, patients receiving radiotherapy <35 days or in a modality other than ≥50 Gray (Gy) beam radiation, and patients with incomplete data	43% (Medium)	TI: median = 30 d	I 39% II 23% III 10% IV 29% No significant association with the treatment interval	5-year overall survival = 60% No significant association with the treatment interval
Liao et al. (40)	2017/2004–2010	Taiwan	18,677	Retrospective cohort	Inclusion: all patients from Taiwan with a diagnosis of oral squamous cell carcinoma (ICD-O-3 codes C00, C02 except C02.4, C03, C04 except C04.9, C05 except C05.1 and C05.2, C06). Exclusion: patients with a previous history of cancer, patients with *in situ* carcinoma, patients with stage IVC disease (i.e., M1 status), patients with treatment interval over 365 days, and patients with incomplete data	71% (Medium)	TI: median = 19 d (IQR = 13–28) in women, median = 19 d (IQR = 13–27) in men	I 28% II 37% III 10% IV 26% Longer treatment intervals were associated with more advanced stages (*p* value of chi-square test<0.001 when TI was categorized into <21, 21–45, 46–90, and > 90 days)	5-year overall survival = 66% Longer treatment intervals were associated with lower survival rates (HR = 1.28 [95%CI = 1.14–1.45] for >90 d compared to <21 d, when TI was categorized into <21, 21–45, 46–90, and > 90 days)
Polesel et al. (41)	2017/2003–2009	Italy	462	Cross-sectional	Inclusion: patients with invasive squamous cell carcinoma of the oral cavity, oropharynx, hypopharynx, and larynx (ICD-10 codes: C00.3-C00.9, C01-C06, C09-C10, C13, C14, C32) Exclusion: cases diagnosed in patients >75 years, cases diagnosed on death certificate only, and patients lost at follow-up	71% (Medium)	TI: median = 28 d	Only available for the total sample (including oropharyngeal, hypopharyngeal, and laryngeal cancer): I 18% II 6% III 6% IV 26% Unknown 42% Association with the treatment interval not studied for oral cancer	5-year overall survival = 58% Longer treatment intervals were associated with lower survival rates (*p*-value of log-rank test <0.01 when TI categorized into <30, 30–44, 45–89, and > 89 days)
Varela-Centelles et al. (42)	2017/2015	Spain	74	Retrospective cohort	Inclusion: patients with a pathologically confirmed incident oral squamous cell carcinoma (ICD codes not specified) recruited at the Oral and Maxillofacial Surgery services of two hospitals in North West Spain Exclusion: recurrent cancer cases, patients with multiple carcinomas, and patients treated outside of the public health service network	71% (Medium)	PI: median = 32 d (IQR = 7–61) DI: median = 36 d (IQR = 12–86) TI: median = 22 d (IQR = 14–33)	I-II 47% III-IV 53% Association with the patient, diagnostic or treatment interval not studied	Not studied
Kerdpon et al. (43)	2017/2011	Thailand	154	Cross-sectional	Inclusion: consecutive patients with oral squamous cell carcinoma in the lip and oral cavity (ICD-10 codes C00-C06) who attended the Head and Neck Tumor Out-patient Clinic, Prince of Songkla University Hospital Exclusion: none	69% (Medium)	PI: median = 30 d (IQR = 7–90) DI: median = 14 d (IQR = 0–47)	Not studied	Not studied
Flukes et al. (44)	2018/2015–2017	Australia	66	Retrospective cohort	Inclusion: Patients with biopsy-confirmed head and neck cancer at any tumor stage and managed at a tertiary referral center. Mucosal subsites included oral, oropharyngeal, sinonasal, nasopharyngeal, laryngeal, hypopharyngeal, and cervical esophagus (ICD-10 codes C00-C15); cutaneous carcinomas were included (ICD-10 codes C44.0-C44.4, C76) Exclusion: patients with thyroid carcinomas	43% (Medium)	TI: mean = 53 d	Only available for the total sample (including oropharyngeal, laryngeal, and cutaneous head and neck cancer): I 14% II 11% III 19% IV 56% Association with the treatment interval not studied for oral cancer	Not studied
Majeed et al. (45)	2018/2016–2017	Pakistan	32	Cross-sectional	Inclusion: patients with cancer (ICD codes not specified) enrolled in Jinnah Hospital Lahore Exclusion: patients who were unable to communicate	21% (High)	PI: median = 90 d (IQR = 45–180) TI: median = 20 d (IQR = 7–32)	Not studied	Not studied
Wang et al. (46)	2018/2007–2010	United States	247	Retrospective cohort	Inclusion: patients ≥18 years diagnosed with histologically confirmed squamous cell carcinoma of the oral cavity (ICD-O-3 codes C02 except C02.4, C03, C04, C05.0, C06.0) in the Kaiser Permanente Northern California healthcare system Exclusion: cases caused by a second primary tumor or a recurrent tumor, patients with an *in situ* carcinoma, patients who were not treated, and patients with incomplete or erroneous data	70% (Medium)	PI: median = 60 d (IQR = 28–281) DI: median = 14 d (IQR = 4–60) TI: median = 27 d (IQR = 18–41)	I 54% II 13% III 13% IV 19% No significant association with the patient, diagnostic, or treatment interval	Not studied
Marella et al. (47)	2018/2000–2016	Italy	59	Retrospective cohort	Inclusion: patients with squamous cell carcinoma of the oral cavity (ICD codes not specified) diagnosed in the University Hospital of Modena and Reggio Emilia Exclusion: none	0% (High)	PI: mean = 113 d (SD = 110)	Not studied	Not studied
Swann et al. (48)	2018/2014	United Kingdom	268*	Retrospective cohort	Inclusion: all incident malignant cancer cases (ICD codes not specified) among England residents for whom their general practices returned data Exclusion: cases of non-melanoma skin cancer	67% (Medium)	DI: median = 39 d (IQR = 17–74)	Not studied	Not studied
Webster et al. (49)	2019/2019	Australia	103	Prospective cohort	Inclusion: patients diagnosed with pathologically verified oral cancer –excluding lip – (ICD codes not specified) through the Royal Brisbane and Women’s Hospital Head and Neck Clinic Exclusion: patients with lip cancer	23% (High)	PI: median = 14 d	Not studied	Not studied
Zhang et al. (50)	2019/2018–2019	China	128	Cross-sectional	Inclusion: patients ≥18 years with pathologically confirmed oral cancer (ICD codes not specified) in the Hospital of Stomatology, at the University of Jilin in China Exclusion: patients with mental disorders, patients with recurrent oral cancer, and patients with oral cancer that was concomitant with other cancers	50% (Medium)	PI: median = 30 d	Not studied	Not studied
Almubarak et al. (51)	2020/2005–2015	Saudi Arabia	61	Cross-sectional	Inclusion: patients with oral cancer (including squamous cell carcinoma, basal cell carcinoma and lymphoma; ICD codes not specified) diagnosed and treated at Asir Central Hospital Exclusion: none	14% (High)	TI: mean = 82 d (SD = 197)	I 3% II 25% III 0% IV 0% Unknown 78% Association with the treatment interval not studied	Not studied
Ganesan et al. (52)	2020/2016–2017	India	119	Retrospective cohort	Inclusion: patients ≥18 years with confirmed head and neck cancer (ICD codes not specified) attending the ear, nose, and throat cancer clinic in a teaching tertiary care setting in Puducherry, South India, and enrolled before treatment initiation Exclusion: patients unable to communicate	54% (Medium)	PI: median = 30 d (IQR = 20–65) DI: median = 30 d (IQR = 15–47)	Only available for the total sample (including oropharyngeal and laryngeal cancer): I 4%, II 12% III 25% IV 60% Association with the patient or diagnostic interval not studied for oral cancer	Not studied
Murchie et al. (53)	2020/2014	United Kingdom	51	Retrospective cohort	Inclusion: all incident cancer cases (ICD codes not specified) in Scotland whose records were analyzed by the general practices that participated in the study audit Exclusion: cases of non-melanoma skin cancer	75% (Low)	DI: median = 35 d (IQR = 13.5–80.5)	Not studied	Not studied
Varela-Centelles et al. (54)	2021/2015–2019	Spain	181	Cross-sectional	Inclusion: incident cases with pathological diagnosis of oral squamous cell carcinoma (ICD codes not specified) diagnosed at two hospitals in Galicia, North-Western Spain. Symptomatic patients and patients whose physical changes or symptoms prompted them to seek care in primary care were included Exclusion: recurrent cancer cases, patients with multiple carcinomas, patients with second primary tumors or metastatic cancer, patients treated at some stage at private clinics, and patients with records of hospital admissions from hospital accident and emergency services	50% (Medium)	PI: median = 31 d (IQR = 7–61) DI: median = 35 d (IQR = 15–82) TI: median = 23 d (IQR = 17–33)	I-II 43% III-IV 57% No significant association with the patient, diagnostic, or treatment interval	Not studied
Jensen et al. (55)	2021/2000–2014	Denmark	862	Retrospective cohort	Inclusion: patients treated for a primary oral cavity squamous cell carcinoma (ICD codes not specified) at Rigshospitalet, Copenhagen University Hospital Exclusion: patients not treated with curative intent and patients with a time interval of 0 days	43% (Medium)	TI: median = 31 d (IQR = 22–43)	I 27% II 20% III 14% IV 29% Unknown 9% Longer treatment intervals were positively correlated with more advanced stages (Spearman’s rho coefficient = 0.16, *p* < 0.001)	Overall survival: not reported Recurrence-free survival: not reported No significant association between overall or recurrence-free survival and the treatment interval
Philip and Kannan (56)	2021/2019–2020	India	261	Cross-sectional	Inclusion: patients with an incident malignant neoplasm of the oral cavity (ICD-10 codes C00-C06) diagnosed at a tertiary cancer center in Kerala, South India Exclusion: recurrent cancer cases, patients with multiple cancers, patients diagnosed through routine cancer surveillance programs, patients who had completed treatment, and patients unwilling to participate	54% (Medium)	PI: median = 92 d (IQR = 38–168)	I-II 32% III-IV 68% Longer patient intervals were associated with more advanced stages (OR = 2.6 [95%CI = 1.3–5.2] for stages III-IV when PI was categorized into ≤90 and > 90 days)	Not studied
Badri et al. (57)	2022/2005–2017	Canada	34*	Qualitative	Inclusion: patients with stage IV oral cancer. The sites included were lip, oral tongue and gum, floor of the mouth, palate and other unspecified parts of the mouth, base of the tongue, tonsil and lingual tonsil, oropharynx, and pharynx otherwise not specified, and Waldeyer’s ring (ICD-O-3 codes C00.3-C00.9, C1-C6, C9-C10, C14) Exclusion: none	60% (Medium)	PI: median = 31 d DI: median = 38 d TI: median = 56 d	Only available for the total sample (including oropharyngeal cancer): IV 100% Association with the patient, diagnostic or treatment interval not studied	Not studied

Asterisks (*) indicate studies in which the sample size included patients with oral and oropharyngeal cancer. d, days; DI, diagnostic interval; ICD, International Classification of Diseases; ICD-O, International Classification of Diseases for Oncology; IQR, interquartile range; N, sample size (i.e., total number of individuals with oral cancer); PI, patient interval; SD, standard deviation; TI, treatment interval.

Overall, the studies included 30,845 patients with oral cancer, of which 1,995 had data on the patient interval, 1,303 on the diagnostic interval, and 29,047 on the treatment interval. In terms of articles, 17 reported data on the patient interval, 11 on the diagnostic interval, 14 on the treatment interval, and 4 on the three intervals of interest simultaneously. The main data sources were medical records (*n* = 15), and questionnaires or interviews (*n* = 14). Most studies were conducted in a hospital or clinical setting (*n* = 19) (Supplementary Data S3).

The average score on the Aarhus checklist was 47%. The risk of bias was high in 7, medium in 19, and low in 2 studies. Most studies provided precise, transparent, and reproducible interval definitions (*n* = 18), 12 fully described the healthcare context, and seven acknowledged the need for theoretical validation or referenced a framework underpinning the measurement of intervals and time points. The individual ratings are shown in Supplementary Table S1.

### 3.2. Meta-analysis

**Figure 2:**
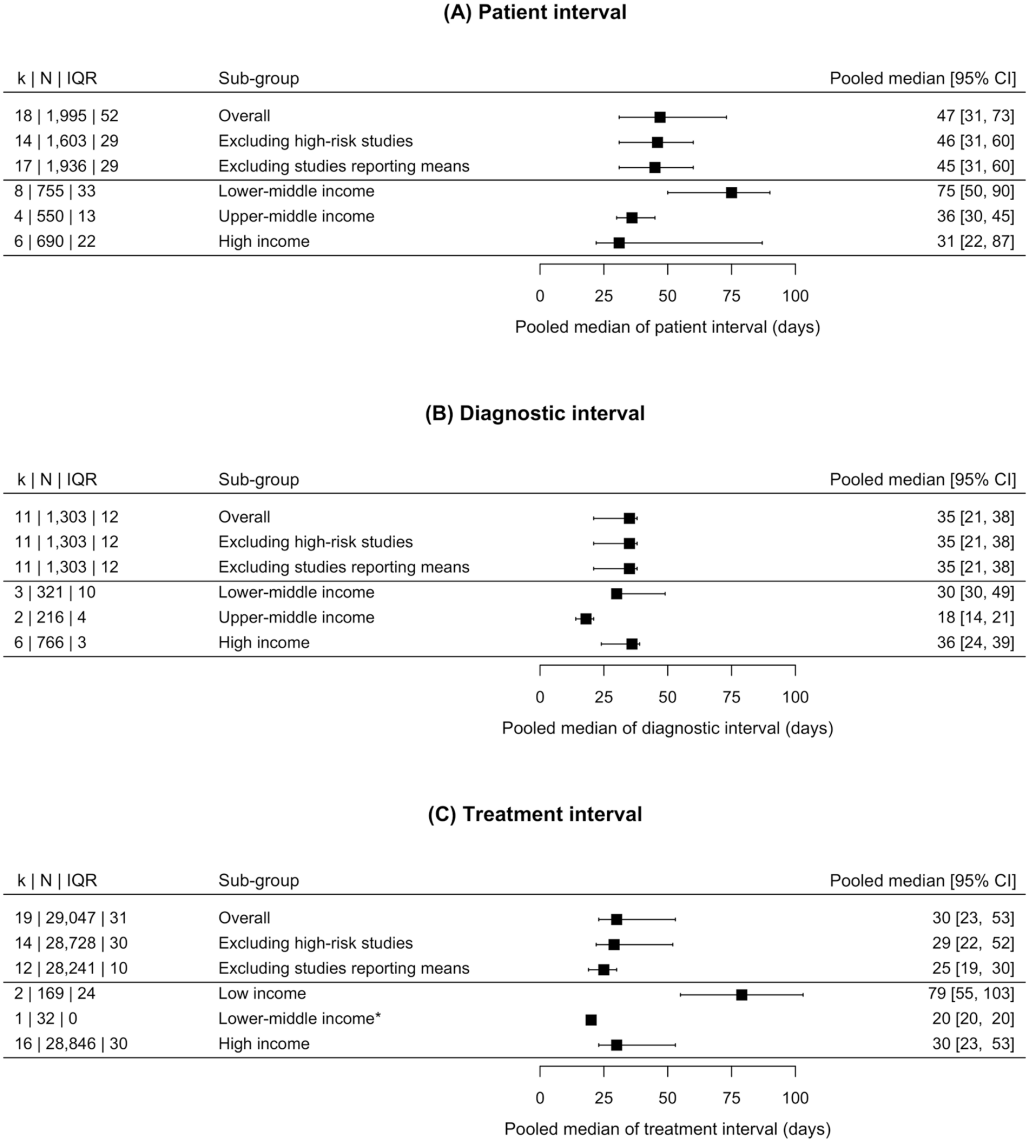
Forest plot representing the pooled median duration and 95% confidence intervals for the patient **(A)**, diagnostic **(B)**, and treatment **(C)** intervals in oral cancer. The asterisk (*) represents a median obtained from only one study, which prevented an estimation of the confidence interval. CI, confidence interval; IQR, interquartile range. k: number of studies or, alternatively, number of groups of patients in whom an interval duration was measured (e.g., a study may have reported the duration of the treatment interval for women and men separately). N: number of patients with available information on each interval’s duration.

Figure 2 illustrates the pooled estimates of the patient, diagnostic, and treatment interval.

#### 3.2.1. Patient interval

The median duration of the patient interval varied widely between studies, ranging from 14 to 92 days. The pooled estimate of all studies was 47 days (95% CI = 31–73), *k* = 18, and it varied only slightly after the exclusion of high-risk studies and studies reporting only means. A gradient by income level was observed for the pooled estimate, such that in lower-middle-income countries, with 75 days (95% CI = 50–90), it was twice as long as in upper-middle-income countries, with 36 days (95% CI = 30–45), and in high-income countries, with 31 days (95% CI = 22–87).

#### 3.2.2. Diagnostic interval

The diagnostic interval showed a smaller variation, its median ranging between 14 and 49 days. The pooled estimate was 35 days (95% CI = 21–38), *k* = 11; all studies analyzing the diagnostic interval had a low or medium risk of bias and reported medians. The diagnostic interval showed no recognizable pattern according to country income level.

#### 3.2.3. Treatment interval

The treatment interval ranged from 18 to 56 days, with an overall pooled estimate of 30 days (95% CI = 23–53), *k* = 19, which was reduced to 25 days (95% CI = 19–30), *k* = 12, after excluding studies that only reported means. The treatment interval was longer in studies from low-income countries (79 days [95% CI = 55–103]) compared to lower-middle-income (20 days in one available study) and high-income countries (30 days [95% CI = 23–53]). There were no studies from upper-middle-income countries reporting the treatment interval.

### 3.3. Stage at diagnosis

The tumor stage at diagnosis was recorded in 19 articles, 6 of which categorized the stage into early (stages I and II) and advanced (stages III and IV); in 5 studies, the stage was reported for patients with oral and other related cancers (usually oropharynx and larynx) together (see Table 1). The proportion of patients in each stage at diagnosis varied broadly between studies (Table 1).

Nine studies analyzed the association between the duration of the intervals of interest and oral cancer stage at diagnosis (see Table 2). The reporting of significant vs. non-significant results did not appear to be related to the study sample size or the risk of bias in studies but was related to the income level of the country.

**Table 2:** Association between the intervals on the oral cancer care pathway, stage and diagnosis, and survival.

	Stage at diagnosis		Survival	
Association	Significant	Non-significant	Significant	Non-significant
Patient interval	2 studies (31, 56)^†^ (intervals >60 and > 90 days, respectively)	3 studies (32, 46, 54)	Not studied	Not studied
Diagnostic interval	1 study (31)^†^ (intervals >30 days)	3 studies (32, 46, 54)	Not studied	Not studied
Treatment interval	2 studies (40, 55)^†^ (longer intervals and intervals >20 days, respectively)	4 studies (37, 39, 46, 54)	3 studies [37,40,41]^††^ (intervals >20, >20 and > 45 days, respectively)	2 studies (39, 55)

^†^Studies finding a significant positive association (i.e., longer intervals were associated with more advanced stages at diagnosis). ^††^Studies finding a significant negative association (i.e., longer intervals were associated with lower survival).

Two studies, conducted in lower-middle-income countries, found that patient intervals longer than 60 days (31) and 90 days (56), respectively, were associated with more advanced stages; however, three studies from high-income countries reported no significant association between the patient interval and stage at diagnosis (32, 46, 54). One study from an upper-middle-income country reported that diagnostic intervals longer than 30 days were associated with more advanced stages (31); nonetheless, three other studies from high-income countries reported no association (32, 46, 54). Finally, the relationship between the treatment interval and stage at diagnosis was only reported in studies from high-income countries. Two studies (40, 55) reported that treatment intervals longer than 20 days were associated with more advanced stages but four other studies reported no significant association (37, 39, 46, 54).

### 3.4. Survival

Survival was analyzed in seven studies, all from high-income countries. The most frequently reported measure was 5-year overall survival, which ranged from 58 to 66%, while one study estimated 2-year overall survival at 86%. Other measures studied included 5-year disease-specific survival (67%), median survival (4.3 years), and recurrence-free survival (not reported).

None of the studies examined the association between the patient or the diagnostic interval and survival. Five studies analyzed the association between the treatment interval and survival. In three of them, treatment intervals longer than 20 days (37, 40) and 45 days (41) were associated with lower survival rates. These were the studies with relatively higher methodological quality, with an average score of 76%. The remaining studies found no significant association (39, 55).

Table 2 summarizes the association between the patient, diagnostic and treatment intervals with stage at diagnosis and survival.

## 4. Discussion

### 4.1. Duration of intervals

To the best of our knowledge, this is the first review to offer pooled meta-analytic estimates of intervals on the oral cancer care pathway using validated methods. Our results suggest that, on average, at least half of patients take more than 1.5 months to consult a healthcare professional after noticing symptoms, and for at least half of patients it takes about a month to have oral cancer diagnosed and another month to start treatment.

The meta-analysis also revealed important differences between high and lower-income countries. All three intervals were relatively homogenous in studies from high-income countries with pooled median duration of about 1 month for each interval. However, in lower-income countries, the patient interval was notably higher (and was associated with more advanced stages at diagnosis). Likewise, the treatment interval was longer in lower-income countries, but the number of studies was much smaller and this finding should therefore be interpreted with caution.

These macro-level findings echo the results of several previous studies showing that socioeconomic factors play a role in oral cancer outcomes. For instance, a systematic review identified economic constraints as a reason for longer patient and diagnostic intervals in oral cancer (58). A relationship between socioeconomic factors and oral cancer survival was suggested in a study conducted in Germany (59). In India, lower socioeconomic status was related to a longer patient interval in patients with oral or oropharyngeal cancer (60). Another study described a direct link between deprivation at the neighborhood level and oral cancer mortality in England (61). Overall, socioeconomic deprivation—across and within countries—may contribute to longer interval duration and indirectly to lower oral cancer survival.

### 4.2. Stage at diagnosis and survival

The analysis of the relationship between the duration of specific intervals and the stage at diagnosis showed inconsistent results: while longer patient and diagnostic intervals were related to later stage at diagnosis, this association was restricted to studies from lower-income countries. Long delays in help-seeking for symptoms, such as those that tend to occur in more socioeconomically deprived contexts, might lead to a more advanced stage at diagnosis. In contrast, the relationship between the duration of the treatment interval and the stage was only examined in studies from high-income countries, showing contradictory results.

In addition, oral cancer stage at diagnosis exhibited great variation. In about half of the studies included, which were conducted in high-income countries, diagnosis at earlier stages (I-II) was more common, whereas, in the remainder, more advanced stages (III-IV) were more frequent. The proportion of patients diagnosed at each stage is context-dependent (62, 63) and intervals are frequently arbitrarily categorized in an attempt to distinguish acceptable intervals from delays. These two factors may be contributing to the mixed results in the literature: in this review, for each of the three intervals of interest, we found at least one study reporting a positive association and at least one study reporting no association at all.

In the current review, 5-year overall survival was approximately 60%, similar to that found in other studies from South Korea (64), Finland, and Sweden (65). In studies from high-income countries with a lower risk of bias, longer treatment intervals were associated with poorer survival rates, in accordance with the systematic review by Graboyes et al. (13). In this vein, an inverse association has been described between the time elapsed from presentation to the start of treatment and survival, at least in early-stage disease (66). However, no study analyzed the impact of the patient or the diagnostic intervals on survival. Overall, more studies are needed to better understand the relationship between the intervals studied and oral cancer survival.

### 4.3. Broader implications for practice

This review suggests that patient intervals in lower-income contexts could be longer than 2 months for the majority of patients and that longer duration of intervals may be associated with later stage at diagnosis. Thus, reducing the time elapsed between symptom start and presentation to a healthcare professional could be an effective strategy to improve patient outcomes. Several factors shape the duration of intervals beyond the socioeconomic context. Arguably, the patient interval is strongly influenced by patient awareness about oral cancer (67, 68). Better knowledge of cancer symptoms and less negative beliefs about cancer are associated with shorter patient intervals (69, 70). Unfortunately, patient awareness about oral cancer, its risk factors, and main warning signs remains low, especially in low- and lower-middle-income countries (71–73); the fact that highly educated individuals are no exception may constitute a matter of concern (74). Furthermore, as stated in the Model of Pathways to Treatment (5), the patient’s journey takes place within a specific healthcare context. Consequently, barriers to healthcare access may also prolong the patient interval (75). A systematic review reported lower access to dental services in individuals without insurance coverage, rural residents, and ethnic minorities (76).

The diagnostic interval is strongly influenced by professional awareness of oral cancer, in which gaps in knowledge, attitude, and practices have been identified in different regions, including low-income countries (perhaps due to insufficient training) (77, 78). Healthcare providers tend to underestimate the importance of betel quid and diet as risk factors and medical and dental practitioners vary in their diagnostic procedures (79). In addition, comorbidity may increase the diagnostic interval (80) and, especially, the treatment interval, as it adds complexity to clinical management. Besides advanced stages at diagnosis (discussed above), other factors germane to longer treatment intervals include transitions in care (81), radiotherapy as the primary treatment (41), and unavailability of timely delivery of care (9).

More broadly, neglect of the social determinants of health and health behavior theory has rendered multiple strategies aimed at raising public and professional awareness of oral cancer ineffective in the long term (82). In particular, media awareness campaigns can increase knowledge about oral cancer but more evidence is needed for their long-term effectiveness in reducing intervals and late-stage disease (82). Two additional approaches could help reduce the burden of oral cancer. Primary prevention includes the reduction of tobacco and alcohol consumption, whereas secondary prevention encompasses systematic screening of high-risk groups (83) and opportunistic screening in primary care (84). In regard to the latter, the dental team plays an essential role. Training of dental students includes risk factors for oral cancer, as well as detection of premalignant lesions (85); in fact, having regular dentist care is associated with earlier stages at diagnosis (86). Nevertheless, high-risk individuals are less likely to be regular dental attendees and to potentially benefit from dental screening (87). Acknowledging that access to dental practitioners is highly dependent on the organization of each country’s healthcare system, we think that a close collaboration between dental and medical professionals in primary care could be a promising way forward.

### 4.4. Limitations

Firstly, the measurement of the intervals studied could be biased in a number of ways (e.g., recall bias), evidenced by the great variation in risk of bias scores according to the Aarhus checklist. Although excluding studies with a high risk of bias had little effect on the pooled estimates, only a few articles were classified as low risk, indicating that the measurement of oral cancer time intervals is not yet sufficiently standardized. Secondly, our findings may be subject to selection bias in the articles included (88), as most studies did not include patients who were too ill to answer, died shortly after diagnosis, presented unusually long intervals, or had missing data. As a result, estimates of interval duration may have limited external validity, despite the global scope of this review. Thirdly, to minimize the risk of publication bias, sources of gray literature were searched and numerous languages were considered. However, tests for publication bias (e.g., Egger’s test), could not be performed as data pooled into meta-analysis is not based on significance testing. Fourthly, although we examined the risk of bias regarding interval measurement, we did not use any of the common tools to assess the broader methodological quality of the studies included [e.g., the Newcastle-Ottawa scale for observational studies (89)]. The objectives of this review were to estimate the duration of intervals on the oral cancer care pathway and to explore potential prognostic implications, rather than measuring the overall quality of extant evidence. Fifthly, because of selective and heterogeneous reporting of the stage at diagnosis and survival, we were unable to perform a quantitative synthesis of their association with interval duration. Sixthly, as expected, high-income countries were vastly more represented than lower-income countries. To adequately inform global policies intending to reduce the burden of oral cancer, more research is needed in low- and middle-income countries, which requires addressing inequities halting global health research (90).

### 4.5. Conclusion

Overall, interval duration is influenced by the socioeconomic context and may have key implications for patient outcomes. In particular, in lower-income countries, patient and treatment intervals were longer, and longer patient intervals were related to later stage at diagnosis. In studies with a smaller risk of bias from high-income countries, longer treatment intervals were associated with lower survival. Further research is needed, particularly in under-resourced settings and in relation to survival.

## Data availability statement

All materials, raw data, and analysis code used in this study are publicly available on the Open Science Framework. This data can be found here: 10.17605/OSF.IO/Y4B6G.

## Author contributions

DP and NFF-M contributed to the conception and design of the study and wrote the first version of the manuscript. NFF-M, DP, and ZŠ performed the article selection and data extraction. MR-B and DP organized the databases and performed the statistical analysis. All authors contributed to manuscript editing and revision, read, and approved the submitted version.

## Funding

This work was supported by the Spanish Association Against Cancer [Asociación Española contra el Cáncer, PROYE20023SANC “High resolution study of social inequalities in cancer (HiReSIC)”], the Cancer Epidemiological Surveillance Subprogram of the CIBER of Epidemiology and Public Health and the Health Institute Carlos III (VICA), and the Health Institute Carlos III (PI18/01593 “Multilevel population-based study of socioeconomic inequalities in the geographical distribution of cancer incidence, mortality and net survival”). DP was supported by a Juan de la Cierva Fellowship from the Ministry of Science and the National Research Agency of Spain (MCIN/AEI, JC2019-039691-I, http://doi.org/10.13039/501100011033, Accessed October 4, 2021). The funders had no role in study design, data collection and analysis, the decision to publish, or preparation of the manuscript.

## Conflict of interest

The authors declare that the research was conducted in the absence of any commercial or financial relationships that could be construed as a potential conflict of interest.

## Publisher’s note

All claims expressed in this article are solely those of the authors and do not necessarily represent those of their affiliated organizations, or those of the publisher, the editors and the reviewers. Any product that may be evaluated in this article, or claim that may be made by its manufacturer, is not guaranteed or endorsed by the publisher.

## Footnotes

^1^ https://www.covidence.org
