# Supplementary material for: The duration of intervals on the oral cancer care pathway and implications for survival: a systematic review and meta-analysis
Source: Front Public Health. 2023 Aug 8;11:1183244. doi: 10.3389/fpubh.2023.1183244 (PMC10442570; doi:10.3389/fpubh.2023.1183244)
Supplement: Supplementary file 1 [file Table_1.docx]

Supplementary Material

The duration of intervals on the oral cancer care pathway and implications for survival: a systematic review and meta-analysis

**Nicolás Francisco Fernández-Martínez^1,2,3^, Dafina Petrova^1,2,3,*^, Zuzana Špacírová^1,2,3^, Rocío Barrios-Rodríguez^1,3,4^, Mario Pérez-Sayáns^5,6^, Luis Miguel Martín-delosReyes^2,3^, Beatriz Pérez-Gómez^3,7^, Miguel Rodríguez-Barranco^1,2,3^, María José Sánchez^1,2,3,4^**

^1^Instituto de Investigación Biosanitaria ibs.GRANADA, Avenida de Madrid, 15, 18012 Granada, Spain

^2^Escuela Andaluza de Salud Pública (EASP), 18011 Granada, Spain

^3^CIBER of Epidemiology and Public Health (CIBERESP), Avenida Monforte de Lemos 3-5, 28029 Madrid, Spain

^4^Department of Preventive Medicine and Public Health, University of Granada, Avenida de la Investigación, 11, 18016 Granada, Spain

^5^Oral Medicine, Oral Surgery and Implantology Unit (MedOralRes), School of Medicine and Dentistry, University of Santiago de Compostela, Rúa de San Francisco, s/n, 15782 Santiago de Compostela, Spain.

^6^ORALRES Group, Health Research Institute of Santiago de Compostela (IDIS), Travesía da Choupana, s/n, 15706 Santiago de Compostela, Spain.

^7^Department of Epidemiology for Chronic Diseases, National Center of Epidemiology, Instituto de Salud Carlos III, C. de Melchor Fernández Almagro, 5, 28029 Madrid, Spain.

*** Correspondence:**Dafina Petrova, PhD
dafina.petrova.easp@juntadeandalucia.es
Escuela Andaluza de Salud Pública. Cuesta del Observatorio, 4. 18011-Granada (Spain)

# Supplementary Data

**Supplementary Data 1. Search strategy.**

MEDLINE (via OVID)

1. exp Neoplasms/

2. neoplasm*.tw.

3. tumo$r:*.tw.

4. carcinoma*.tw.

5. cancer*.tw.

6. oncolog*.tw.

7. or/1-6

8. patient delay.tw.

9. help-seeking Interval.tw.

10. patient interval.tw.

11. (time to help-seeking or time to help seeking).tw.

12. (help-seeking delay or help seeking delay).tw.

13. (time to first presentation or time to first consultation).tw.

14. (appraisal interval or appraisal delay).tw.

15. (diagnos* interval or diagnos* delay).tw.

16. (provider delay or system delay or referral delay or investigation delay).tw.

17. (system interval or doctor interval or physician interval or referral interval or primary care interval).tw.

18. (treatment interval or treatment delay or pre-treatment interval or pre-treatment delay).tw.

19. (scheduling delay or scheduling interval).tw.

20. (therapeutic delay or therapeutic interval).tw.

21. *"Appointments and Schedules"/

22. or/8-21

23. 7 and 22

24. (animals not humans).tw.

25. 23 not 24

26. limit 25 to yr="2009 -Current"

Of note, this search strategy was then translated to other data sources, fundamentally Embase and Web of Science.

**Supplementary Data 2. Aarhus statement checklist (short version).**

The checklist was adapted for the review in a team meeting: several items were dropped due to inapplicability (e.g., questions regarding referral intervals), some questions were further clarified, and common criteria were set. The final checklist contained 15 items: 5 applied to all studies and 10 applied as a function of the interval measured or data source used. The answer options for each question were “yes”, “no”, “uncertain”, or “not applicable”. The checklist was completed independently by two reviewers and disagreements were resolved by a third reviewer. One point was awarded for each “yes” response (“no” and “uncertain” received 0 points each) and the proportion of “yes” responses out of the total that applied was calculated as a measure of risk of bias, resulting in a score ranging from 0% to 100%. Studies with <25% were considered high risk and studies with ≥75% low risk, with the rest considered intermediate.

Response options for each dimension:

If the answer is YES: Low risk of bias

If the answer is PARTIALLY: Uncertain

If the answer is NO: High risk of bias

Each YES gives 1 point towards a final Methodological quality score.

|  | **Question** | **Hints:** |
| --- | --- | --- |
| **For all studies:** | 1. Are the beginning and end points of each interval clearly defined? | This must be done for all intervals measured. |
|  | 2. For all time points and intervals described, are there precise, transparent and repeatable definitions? | Must be true for all time points and intervals measured |
|  | 3. Is the healthcare context in which the study is based fully described? | e.g., description of hospitals and their populations, basic description of the usual diagnostic pathway, etc. |
|  | 4. Do the questions on time points and/or intervals clearly derive from stated definitions? | Is there correspondence between the definitions used and what was measured? |
|  | 5. Do researchers acknowledge the need for theoretical validation and/or make reference to the theoretical framework(s) underpinning measurement and analysis of the time points? | e.g., is any of the models (Andersen, Olesen, Arhus statement) mentioned? Is there any other mention of the need for a theoretical model? |
| **For studies that require an estimate of the date of first symptom** | 6. Is there a discussion of the different biases influencing measurement of this time point? |  |
| **For studies that require measurement of a date of first presentation to healthcare** | 7. Do the researchers discuss the complexity of the date of first  presentation? |  |
| **For studies that require measurement of the date of diagnosis** | 8. Do the researchers use an existing hierarchical rationale for the  date of diagnosis measurement? |  |
| **For studies using questionnaires and/or interviews with patients and/or health-care**  **providers** | 9. Has a validated instrument been used? |  |
|  | 10. Is there some discussion of how reliability and validity (trustworthiness) has been established? |  |
|  | 11. Is there discussion of the different biases influencing measurement of the time points, such as how and when the question is asked and who is being asked? |  |
|  | 12. Is the timing of the interview in relation to the date of diagnosis provided? |  |
|  | 13. Is there any triangulation of self-reported data with other data sources such as case notes? |  |
| **For studies using primary case-note/medical record audit:** | 14. Is there a clear and precise description of how case-note/medical record data were used to ascertain time points, and/or with an acknowledgment of limitations of such data? |  |
| **For database analysis:** | 15. Is there a thorough description of the database chosen including sampling coverage and completeness  of information? |  |

**Supplementary Data 3. Detailed information about included articles.**

This information is available on the Open Science Framework (DOI: <10.17605/OSF.IO/Y4B6G>)

# Supplementary Figures and Tables

## Supplementary Tables

**Supplementary Table 1. Aarhus checklist ratings of individual studies included in the review.**

The 15 items in the short form of the Aarhus statement checklist are listed in Supplementary Material S2. The answer options for each question were “yes”, “no”, “uncertain”, or “not applicable”. One point was awarded for each “yes” response (“no” and “uncertain” received 0 points each). The proportion of “yes” responses out of the total that applied ranged from 0% to 100% and was used as an estimate of the risk of bias. Studies with a score below 25% were considered as high risk, studies with a score of 75% and above as low risk, and the rest were considered as intermediate risk.

| **First author /  year of publication** | **Q1** | **Q2** | **Q3** | **Q4** | **Q5** | **Q6** | **Q7** | **Q8** | **Q9** | **Q10** | **Q11** | **Q12** | **Q13** | **Q14** | **Q15** | **#Yes** | **#No** | **#U** | **#NA** | **Score** | **Risk of bias** |
| --- | --- | --- | --- | --- | --- | --- | --- | --- | --- | --- | --- | --- | --- | --- | --- | --- | --- | --- | --- | --- | --- |
| Gao 2009 [30] | Yes | Yes | Yes | U | No | Yes | No | No | No | No | Yes | No | U | NA | NA | 5 | 6 | 2 | 2 | 38% | Medium |
| Sargeran 2009 [31] | Yes | Yes | No | Yes | No | Yes | Yes | No | No | Yes | Yes | Yes | Yes | U | NA | 9 | 4 | 1 | 1 | 64% | Medium |
| Teppo 2009 [32] | Yes | Yes | Yes | Yes | No | Yes | No | No | NA | NA | NA | NA | NA | Yes | NA | 6 | 3 | 0 | 6 | 67% | Medium |
| Kolude 2013 [33] | Yes | No | No | U | No | NA | NA | No | NA | NA | NA | NA | NA | No | NA | 1 | 5 | 1 | 8 | 14% | High |
| Esmaelbegi 2014 [34] | Yes | No | No | U | No | No | No | NA | No | No | No | Yes | No | NA | NA | 2 | 9 | 1 | 3 | 17% | High |
| Zhang 2015 [35] | Yes | U | Yes | U | No | NA | NA | No | NA | NA | NA | NA | NA | No | Yes | 3 | 3 | 2 | 7 | 38% | Medium |
| Baishya 2015 [36] | Yes | No | No | U | No | Yes | No | NA | No | No | No | Yes | No | NA | NA | 3 | 8 | 1 | 3 | 25% | Medium |
| Chiou 2016 [37] | Yes | Yes | Yes | Yes | No | NA | NA | Yes | NA | NA | NA | NA | NA | NA | Yes | 6 | 1 | 0 | 8 | 86% | Low |
| Kaing 2016 [38] | Yes | No | No | U | No | NA | NA | No | NA | NA | NA | NA | NA | No | NA | 1 | 5 | 1 | 8 | 14% | High |
| Fujiwara 2017 [39] | Yes | No | Yes | U | No | NA | NA | No | NA | NA | NA | NA | NA | NA | Yes | 3 | 3 | 1 | 8 | 43% | Medium |
| Liao 2017 [40] | Yes | Yes | Yes | Yes | No | NA | NA | U | NA | NA | NA | NA | NA | NA | Yes | 5 | 1 | 1 | 8 | 71% | Medium |
| Polesel 2017 [41] | Yes | Yes | No | Yes | No | NA | NA | Yes | NA | NA | NA | NA | NA | NA | Yes | 5 | 2 | 0 | 8 | 71% | Medium |
| Varela-Centelles 2017 [42] | Yes | Yes | No | Yes | Yes | Yes | Yes | No | No | No | Yes | Yes | Yes | Yes | NA | 10 | 4 | 0 | 1 | 71% | Medium |
| Kerdpon 2017 [43] | Yes | Yes | Yes | Yes | Yes | Yes | No | No | No | Yes | Yes | No | Yes | NA | NA | 9 | 4 | 0 | 2 | 69% | Medium |
| Flukes 2018 [44] | Yes | Yes | Yes | U | No | NA | NA | No | No | NA | NA | NA | NA | No | NA | 3 | 3 | 1 | 8 | 43% | Medium |
| Majeed 2018 [45] | Yes | Yes | No | Yes | No | No | No | No | No | No | No | No | No | No | NA | 3 | 11 | 0 | 1 | 21% | High |
| Wang 2018 [46] | Yes | Yes | U | Yes | No | Yes | No | Yes | NA | NA | NA | NA | NA | Yes | Yes | 7 | 2 | 1 | 5 | 70% | Medium |
| Marella 2018 [47] | No | No | No | U | No | No | No | NA | NA | NA | NA | NA | NA | No | NA | 0 | 7 | 1 | 7 | 0% | High |
| Swann 2018 [48] | Yes | Yes | Yes | Yes | No | NA | Yes | No | No | No | Yes | Yes | NA | Yes | NA | 8 | 4 | 0 | 3 | 67% | Medium |
| Webster 2019 [49] | Yes | No | No | U | No | No | No | No | No | Yes | No | Yes | No | NA | NA | 3 | 9 | 1 | 2 | 23% | High |
| Zhang 2019 [50] | Yes | Yes | No | Yes | No | Yes | Yes | NA | No | No | Yes | No | No | NA | NA | 6 | 6 | 0 | 3 | 50% | Medium |
| Almubarak 2020 [51] | U | U | No | Yes | No | NA | NA | No | NA | NA | NA | NA | NA | No | NA | 1 | 4 | 2 | 8 | 14% | High |
| Ganesan 2020 [52] | Yes | Yes | Yes | Yes | Yes | Yes | No | No | No | No | No | Yes | No | NA | NA | 7 | 6 | 0 | 2 | 54% | Medium |
| Murchie 2020 [53] | Yes | Yes | Yes | Yes | Yes | NA | Yes | No | No | No | Yes | Yes | NA | Yes | NA | 9 | 3 | 0 | 3 | 75% | Low |
| Varela-Centelles 2021 [54] | Yes | No | No | Yes | Yes | Yes | No | No | No | Yes | Yes | No | Yes | No | NA | 7 | 7 | 0 | 1 | 50% | Medium |
| Jensen 2021 [55] | Yes | Yes | No | Yes | No | NA | NA | No | NA | NA | NA | NA | NA | NA | U | 3 | 3 | 1 | 8 | 43% | Medium |
| Philip 2021 [56] | Yes | Yes | No | Yes | Yes | U | No | NA | Yes | No | Yes | No | Yes | No | NA | 7 | 5 | 1 | 2 | 54% | Medium |
| Badri 2022 [57] | Yes | Yes | Yes | Yes | Yes | No | No | No | NA | NA | NA | NA | NA | Yes | U | 6 | 3 | 1 | 5 | 60% | Medium |

#: Number of items of the Aarhus statement checklist. NA: Not applicable. U: Uncertain.
